# Divergent effects of switching from cytology to HPV-based screening in the Nordic countries

**DOI:** 10.1093/eurpub/ckad225

**Published:** 2024-01-22

**Authors:** Veli-Matti Partanen, Joakim Dillner, Ameli Tropé, Ágúst Ingi Ágústsson, Stefan Lönnberg, Sirpa Heinävaara, Ahti Anttila

**Affiliations:** Finnish Cancer Registry, Helsinki, Finland; Department of Clinical Science, Intervention and Technology - CLINTEC, Karolinska Institutet, Stockholm, Sweden; Section for Cervical Cancer Screening, Cancer Registry of Norway, Oslo, Norway; Icelandic Cancer Screening Coordination Center, Reykjavík, Iceland; Finnish Cancer Registry, Helsinki, Finland; Finnish Cancer Registry, Helsinki, Finland; Finnish Cancer Registry, Helsinki, Finland

## Abstract

**Background:**

Cervical cytology has been the primary method of cervical cancer screening for decades. Tests that detect viral HPV are shown in several randomized trials to provide better protection against cancer compared with cytology. HPV-based screening has been implemented alongside cytology in the Nordic countries for several years. The aim of this study was to compare cytology and HPV-based screening in the colposcopy referrals and detection rates of cervical lesions.

**Methods:**

Individual-level screening data from Finland, Iceland, Norway and Sweden were harmonized and aggregated locally. We utilized data for tests taken during years 2015–17 and biopsies taken during years 2015–19 to allow 24 months of follow-up. Age-standardized estimates and age-adjusted risk ratios for six different outcomes of screening management were calculated.

**Results:**

The age-standardized colposcopy rates were higher in HPV-based testing compared with cytology in Finland (3.5% vs. 0.9%) and Norway (6.0% vs. 4.1%) but lower in Sweden (3.7% vs. 4.9%). The relative detection rate of cervical intraepithelial neoplasia grade 2 and above in HPV-based testing compared with cytology was highest in Finland (RR 2.37, 95% CI 2.13–2.63) and Norway (RR 1.66, 95% CI 1.57–1.72) while in Sweden the difference was not statistically significant (RR 0.98, 95% CI 0.95–1.00).

**Conclusions:**

The effects of implementing HPV screening varied by country as different screening algorithms were implemented. HPV-based screening increases colposcopy rates mainly through referrals from increased repeat testing and detection rate is therefore significantly higher compared with cytology. Monitoring of these indicators in subsequent rounds of HPV-based screening remains essential.

## Introduction

Cervical cancer is globally the fourth most common cancer among women, but the burden is much lower in most developed countries due to screening.^1^ The World Health Organization has committed to eliminating cervical cancer by setting global targets for adequate coverage of vaccination, screening and treatment.^2^ Screening has reduced morbidity and mortality from cervical cancer in the Nordic countries by 60–85% compared with a situation without screening.^3–6^ In Europe, mortality reduction for women attending screening is estimated to range between 41% and 92%.^7^ Within the Nordic countries, incidence of cervical cancer is lowest in Finland, even though the prevalence of oncogenic human papillomavirus (HPV) infection is currently similar compared with Norway and Sweden.^6^^,^^8^ HPV vaccination programmes will further reduce the risk of cervical cancer significantly.^9^^,^^10^ The European Union’s Beating Cancer Plan facilitates this by supporting HPV vaccination programmes of member states.^11^ Nevertheless, screening will remain the main preventive strategy for non-vaccinated women.

Cervical cytology has been the primary test method in the screening programmes for decades. Several randomized trials have shown that high-risk HPV (hrHPV) testing provides better protection against cancer compared with cytology-based screening.^12^ HPV-based screening has been implemented in the Nordic countries alongside cytology for several years, even though Sweden and Norway have currently finished implementation of primary HPV-based screening. This overlap of both HPV tests and cytology in screening during recent years enables the comparison in a routine setting.

The implementation of HPV-based screening will cause changes in the management of women in the programme. The probability of positive test results is higher in HPV testing compared with cytology.^8^ In most cases, however, only the HPV test is positive, and no cervical abnormalities are found in cytology triage. The screening algorithms differ between the Nordic countries leading to different resource needs in the precancer diagnostic and treatment services. The concurrent use of both test methods within countries provides an opportunity to study these differences in a population-based screening setting. This is crucial for making informed decisions on optimal screening algorithms.

In this study, we compared differences in the colposcopy referrals and the detection rates (DRs) of cervical lesions between cytology and HPV-based screening, and between countries.

## Methods

### Screening programmes

All Nordic countries have population-based screening programmes with minor differences on target age groups and screening intervals. A detailed overview of the programmes is available elsewhere.^13^Table 1 provides a summary of the programmes and screening algorithms during the study. In primary cytology testing, HPV test was used as a triage test in Iceland, Norway and Sweden whereas in primary HPV testing, cytology was used as a triage test in all countries.

**Table 1:** Cervical cancer screening programmes in the Nordic countries included in the study during 2015–17

	Finland	Iceland	Norway	Sweden
Target age group (years)	30–60	23–65	25–69	23–64
Screening interval (years)	5	3	3 (25–33)	3 (23–50)
			5 (34–69)	7 (51–64)
Primary screening test	Cytology or HPV test depending on municipality	Cytology (23–64)	Cytology (25–33)	Cytology (23–29)
		HPV test (65)	HPV test (34–69)	HPV test (30–64)
Criteria for repeat testing in cytology-based screening	ASC-US^a^	ASC-US or LSIL in combination with a positive reflex triage HPV test	ASC-US or LSIL in combination with a positive reflex triage HPV test	ASC-US or LSIL with hrHPV negativity for women aged 51–64^b^
Criteria for repeat testing in HPV-based screening	hrHPV positivity with ASC-US or no cervical abnormality in reflex cytology	–	hrHPV positivity with ASC-US or LSIL or no cervical abnormality in reflex cytology	hrHPV positivity with no cervical abnormality in reflex cytology^c^
Criteria for referral to diagnostic confirmation in cytology-based screening	LSIL+/AGC-NOS+ and persistent ASC-US^a^	ASC-H+/AGC+ and ASC-US, LSIL, or hrHPV positivity results that are persistent	ASC-H+/AGC+ and ASC-US, LSIL, or hrHPV positivity results that are persistent	ASC-H+/AGC+ and ASC-US or LSIL with hHPV positivity
Criteria for referral to diagnostic confirmation in HPV-based screening	hrHPV positivity with LSIL+/AGC-NOS+ cytology triage or persistent hrHPV positivity	–	hrHPV positivity with cervical abnormality in cytology triage or persistent hrHPV positivity	hrHPV positivity with cervical abnormality in cytology triage

a Repeat testing was also recommended to under 30-year-old women with LSIL.

b Repeat test interval was 3 years, which corresponded to normal screening interval for women aged 23–50.

c hrHPV-positive women were referred to diagnostic confirmation if the result of the repeat HPV test after 3 years was positive.

### Introduction of HPV-based screening

Norway introduced HPV testing in a randomized setting in 2015 and is currently screening all women over 33 years with an HPV test. In Sweden, the official recommendation by the National Board of Health and Welfare from 2015 states that women aged 30 or older should be screened with an HPV test, but some counties delayed the implementation. The number of HPV tests exceeded cytology-based tests in 2018 and currently all women in the screening programme are screened with a primary HPV test. In Finland, HPV-based screening was used in 2003–14 in a large, randomized study^14^ and the use of HPV tests exceeded cytology-based tests in 2019.^15^ In Iceland, cytology was the primary screening method until the start of 2021, when HPV testing became the primary test method for women aged 30–64 years.

### Data sources

The data have been gathered within the NordScreen project and the process has been described in previous publications.^8^^,^^16^ Briefly, individual-level screening data from Finland, Iceland, Norway and Sweden were harmonized and aggregated locally to standard format. The Finnish data included cervical tests and histological diagnoses taken only within the screening programme whereas other countries had data on all tests and histological results. Cervical test and histological data from Denmark were not available for this study.

We utilized data for tests taken during years 2015–17 and biopsies taken during years 2015–19 to allow 24 months of follow-up for all tests. This enabled information on the diagnostic confirmation due to the primary test result, any reflex triage, as well as from the potential repeat testing.

### Harmonization of data

Test observations within 90 days from the first test were combined into test episodes defined by the most severe cytology and HPV test result. The 90-day interval was used to link the results of a primary HPV test and potential reflex cytology. As in our previous study,^8^ we limited our analysis to primary testing and excluded repeat test results and results of tests of cure. The primary tests were recognized by including only test episodes where the preceding test result within 2.5 years was normal or hrHPV negative, or there were no preceding tests within the last 2.5 years.

The test results were converted from Bethesda or Papanicolaou classification to categories of ‘intermediate positive’ and ‘clearly positive’. Intermediate positive results included atypical squamous intraepithelial lesions (ASC-US), low-grade squamous intraepithelial lesions (LSIL) and HPV-positive test results without any cytological abnormalities in reflex triage. In most cytology-based programmes, these lead to repeat testing; a referral to diagnostic confirmation by colposcopy and biopsy takes place only if such a finding is repeated or progressed to clearly positive result (see below) in the respective repeat testing.^17^^,^^18^ Clearly positive results included high-grade squamous intraepithelial lesions (HSILs) [atypical squamous cells, cannot exclude HSIL (ASC-H) or worse] and glandular lesions [atypical glandular cells (AGC) or worse] denoted as ASC-H+/AGC+, which generally warrant an immediate referral to/for diagnostic confirmation (according to the current screening algorithms). Finland was the only country that divided AGC into not otherwise specified (AGC-NOS) or favouring neoplasia (AGC-FN). There were some differences, e.g. in Sweden a referral to colposcopy and biopsy was done after a primary ASC-US cytology if the HPV triage was positive (table 1). In Finland, guidelines allowed either immediate referral or follow-up testing within 6 months after an AGC-NOS cytology.

Test episodes were linked with histological data by determining the most severe histology, if any, within the follow-up time. We used a registered histological result as a proxy for colposcopy since according to guidelines a biopsy should be taken in a colposcopy. Only the test episode with the most severe result during one calendar year per woman was included in the analyses.

### Statistical analysis

Test episodes of women aged 30–64 years were included in the analyses as HPV testing in Finland and Sweden started at 30 years. For Norway, women aged 30–64 years were also included even though primary HPV testing started at 34 years. All test episodes were grouped into 5-year age groups.

The number of colposcopies and the number of cervical intraepithelial neoplasia grade 2 and above (CIN2+) cases found were calculated for follow-up times of 12 and 24 months. Twenty-four-month follow-up time was also used for calculating the positive predictive value (PPV) of colposcopy for CIN2+ after clearly positive test result, the DR of CIN2+ and the number of colposcopies needed per one found CIN2+ case. Follow-up of 24 months was used to maximize the accumulating number of histological diagnoses from a period corresponding to the current screening round while avoiding the inclusion of histological diagnoses from the next primary testing episode.

We calculated estimates for six different outcomes of screening management using direct age-standardization and stratifying by test method. NORDCAN population was used as the standard population. The 95% confidence intervals (95% CIs) for age-standardized rates were calculated using the binomial exact method.

The outcomes were (i) test positivity, (ii) colposcopy within 12 months, (iii) colposcopy within 24 months, (iv) PPV of colposcopy for CIN2+ after a clearly positive test result, (v) DR for CIN2+ per 1000 primary tests and (vi) number of colposcopies per CIN2+ case.

Test positivity was defined as the proportion of hrHPV+ test results and/or abnormal cytology results out of all primary test episodes. Colposcopies within either 12 or 24 months were calculated as the proportion of women with a histological result registered within follow-up time out of all primary test episodes. PPVs were calculated as the percentage of women with CIN2+ results out of women clearly positive test result and with a histological diagnosis within follow-up. The DR was calculated as the proportion of CIN2+ results out of all primary test episodes. Finally, the number of colposcopies per CIN2+ case was calculated by dividing the number of histological results by the number of CIN2+ results and limiting our analysis to primary tests with any positive result.

We also calculated risk ratios (RRs) for HPV testing compared with cytology separately for Finland, Norway and Sweden for these outcomes using Poisson regression and adjusting for age (5-year age groups). Since HPV testing in Norway starts at age 34 years, as a sensitivity analysis, we also calculated the RRs for women aged 34–64 years.

All data management and analyses were done with R version 3.6.1.

## Results

During years 2015–17 there were a total of 2 538 715 primary test episodes in Finland, Iceland, Norway and Sweden among women aged 30–64 years. Most women were tested with cytology; 87% in Finland, 89% in Norway and 82% in Sweden while all women in Iceland were tested with cytology. Conversely, the proportion of HPV tests in Finland, Norway and Sweden varied between 11% and 18%.

Table 2 shows the results of the primary tests by test method, the number of colposcopies, and CIN2+ cases found within follow-up of 12 and 24 months. Over 90% of test episodes were classified as normal or negative with both test methods and the percentage of clearly positive test results (0.9–1.2% depending on country) were similar regardless of test method. The percentage of women undergoing colposcopy varied considerably both between countries and between test methods. In cytology-based screening in Finland, 0.9% of women had a colposcopy within 24 months while in Norway and Sweden 4.1% and 4.9% of women, respectively, had a colposcopy.

**Table 2:** Summary of cervical test data and histological data in the Nordic countries included in the study from 2015–17 of 30- to 64-year-old women

	Finland		Iceland		Norway		Sweden	
	***N***	**%**	***N***	**%**	***N***	**%**	***N***	**%**
Primary cytology testing	458 095	100.0	51 098	100.0	827 667	100.0	1 201 855	100.0
Missing (%)	19	0.0	0	0.0	0	0.0	93	0.0
Unsatisfactory (%)	41	0.0	52	0.1	10 536	1.3	4326	0.4
Normal or negative (%)	435 616	95.1	46 253	90.5	773 745	93.5	1 140 860	94.9
Intermediate positive (%)	18 395	4.0	4200	8.2	34 117	4.1	46 310	3.9
Clearly positive (%)	4024	0.9	593	1.2	9269	1.1	10 266	0.9
Colposcopies within 12 months^a^	3571 (3490)	0.8 (0.8)	1120 (1094)	2.2 (2.1)	26 540 (16 376)	3.2 (2.0)	48 759 (27 690)	4.1 (2.3)
Colposcopies within 24 months^a^	4033 (3899)	0.9 (0.9)	1264 (1205)	2.5 (2.4)	34 022 (18 673)	4.1 (2.3)	59 140 (30 301)	4.9 (2.5)
CIN2+ cases within 12 months^a^	1337 (1335)	0.3 (0.3)	417 (416)	0.8 (0.8)	8284 (8002)	1.0 (1.0)	10 893 (10 457)	0.9 (0.9)
CIN2+ cases within 24 months^a^	1491 (1477)	0.3 (0.3)	466 (457)	0.9 (0.9)	10 347 (9308)	1.3 (1.1)	12 676 (11 675)	1.1 (1.0)

	*N*	%	*N*	%	*N*	%	*N*	%

Primary HPV testing	67 465	100.0			99 017	100.0	265 528	100.0
Missing (%)	0	0.0			0	0.0	0	0.0
Unsatisfactory (%)	0	0.0			458	0.5	87	0.0
Normal or negative (%)	62 220	92.2			91 657	92.6	241 514	91.0
Intermediate positive (%)	4597	6.8			5838	5.9	21 362	8.0
Clearly positive (%)	648	1.0			1064	1.1	2565	1.0
Colposcopies within 12 months^a^	807 (717)	1.2 (1.1)			3916 (2835)	4.0 (2.9)	8260 (6519)	3.1 (2.5)
Colposcopies within 24 months^a^	2174 (2083)	3.2 (3.1)			5665 (4153)	5.7 (4.2)	9914 (7118)	3.7 (2.7)
CIN2+ cases within 12 months^a^	258 (257)	0.4 (0.4)			1225 (1208)	1.2 (1.2)	2571 (2557)	1.0 (1.0)
CIN2+ cases within 24 months^a^	474 (473)	0.7 (0.7)			1708 (1661)	1.7 (1.7)	2902 (2860)	1.1 (1.1)

	*N*	%	*N*	%	*N*	%	*N*	%

Either	525 560	100.0	51 098	100.0	926 684	100.0	1 476 281	100.0
Missing (%)	19	0.0	0	0.0	0	0.0	93	0.0
Unsatisfactory (%)	41	0.0	52	0.1	10 994	1.2	4428	0.3
Normal or negative (%)	497 836	94.7	46 253	90.5	865 402	93.4	1 390 809	94.2
Intermediate positive (%)	22 992	4.4	4200	8.2	39 955	4.3	68 110	4.6
Clearly positive (%)	4672	0.9	593	1.2	10 333	1.1	12 841	0.9
Colposcopies within 12 months^a^	4378 (4207)	0.8 (0.8)	1120 (1094)	2.2 (2.1)	30 456 (19 211)	3.3 (2.1)	57 403 (34 468)	3.9 (2.3)
Colposcopies within 24 months^a^	6207 (5982)	1.2 (1.1)	1264 (1205)	2.5 (2.4)	39 687 (22 826)	4.3 (2.5)	69 520 (37 700)	4.7 (2.6)
CIN2+ cases within 12 months^a^	1595 (1592)	0.3 (0.3)	417 (416)	0.8 (0.8)	9509 (9210)	1.0 (1.0)	13 546 (13 095)	0.9 (0.9)
CIN2+ cases within 24 months^a^	1965 (1950)	0.4 (0.4)	466 (457)	0.9 (0.9)	12 055 (10 969)	1.3 (1.2)	15 676 (14 631)	1.1 (1.0)

a The number of colposcopies and CIN2+ cases after a positive primary test are marked in brackets.

Clearly positive test results were almost always followed up with colposcopy within a year, except for Finland where the colposcopy percentage was below 85% even after 3 years from primary test (Supplementary figure S1). Upon further analysis (Supplementary figure S2), AGC-NOS results were often not followed up with colposcopy. Intermediate positive results were followed up with colposcopy more often in HPV testing compared with cytology in Finland and Norway, but less often in Sweden. Women with normal cytology or hrHPV negative test results were rarely followed up with colposcopy especially in Finland and Iceland. The proportion of colposcopies after a negative test result out of all colposcopies in cytology-based testing was 44% in Norway and 49% in Sweden (26% and 27% in HPV-based testing, respectively), while the proportions in Finland and Iceland were around 3–5%.

Age-standardized estimates for seven different screening outcomes are shown in table 3. The colposcopy rates within 24 months were higher in HPV-based testing compared with cytology in Finland (3.5% vs. 0.9%) and Norway (6.0% vs. 4.1%) but lower in Sweden (3.7% vs. 4.9%). The DR of CIN2+ was lowest in Finland (3.6 and 8.8 per 1000 primary tests in cytology and HPV testing, respectively) and highest in Norway (12.3 and 19.8, respectively). The number of colposcopies needed for finding a CIN2+ case in cytology-based screening was lowest in Norway (2.3 colposcopies) and highest in Iceland (3.1 colposcopies). There was also considerable difference in Finland between test methods as 2.9 colposcopies were needed on average for a CIN2+ case in cytology while 5.0 were needed in HPV-based screening.

**Table 3:** Different outcomes of screening for women aged 30–64 years during years 2015–17 with 95% CIs. Age-standardized to NORDCAN population

Outcome	Finland		Iceland		Norway		Sweden	
	Cytology	HPV test	Cytology	HPV test	Cytology	HPV test	Cytology	HPV test
Test positivity	5.1 (5.0–5.1)	9.0 (8.7–9.3)	9.5 (9.2–9.8)	–	5.2 (5.2–5.3)	7.5 (7.3–7.7)	4.6 (4.6–4.7)	8.8 (8.7–8.9)
Colposcopy within 12 months	0.8 (0.8–0.9)	1.4 (1.2–1.5)	2.2 (2.1–2.4)	–	3.2 (3.2–3.2)	4.2 (4.0–4.3)	4.0 (4.0–4.1)	3.0 (3.0–3.1)
Colposcopy within 24 months	0.9 (0.9–1.0)	3.5 (3.3–3.6)	2.5 (2.4–2.7)	–	4.1 (4.0–4.1)	6.0 (5.8–6.2)	4.9 (4.8–4.9)	3.7 (3.6–3.7)
PPV for CIN2+ after a clearly positive test result	42.5 (40.1–45.1)	37.0 (31.5–43.3)	49.4 (42.7–57.3)	–	63.3 (61.5–65.2)	65.2 (59.6–71.3)	67.1 (65.3–69.0)	69.4 (65.5–73.6)
Detection rate of CIN2+ per 1000 primary tests	3.6 (3.4–3.8)	8.8 (7.9–9.9)	9.3 (8.5–10.2)	–	12.3 (12.1–12.6)	19.8 (18.6–21.0)	10.1 (9.9–10.3)	10.3 (10.0–10.7)
Number of colposcopies per CIN2+ case	3.0 (2.9–3.1)	5.2 (5.0–5.4)	3.2 (2.9–3.4)	–	4.6 (4.5–4.6)	3.8 (3.7–4.0)	6.3 (6.2–6.3)	4.8 (4.6–4.9)
Number of colposcopies per CIN2+ case^a^	2.9 (2.8–3.0)	5.0 (4.8–5.2)	3.1 (2.9–3.3)		2.3 (2.2–2.3)	2.7 (2.6–2.8)	3.0 (3.0–3.0)	3.0 (2.9–3.1)

a Only colposcopies and CIN2+ cases after any positive primary test result were considered.

**Figure 1:**
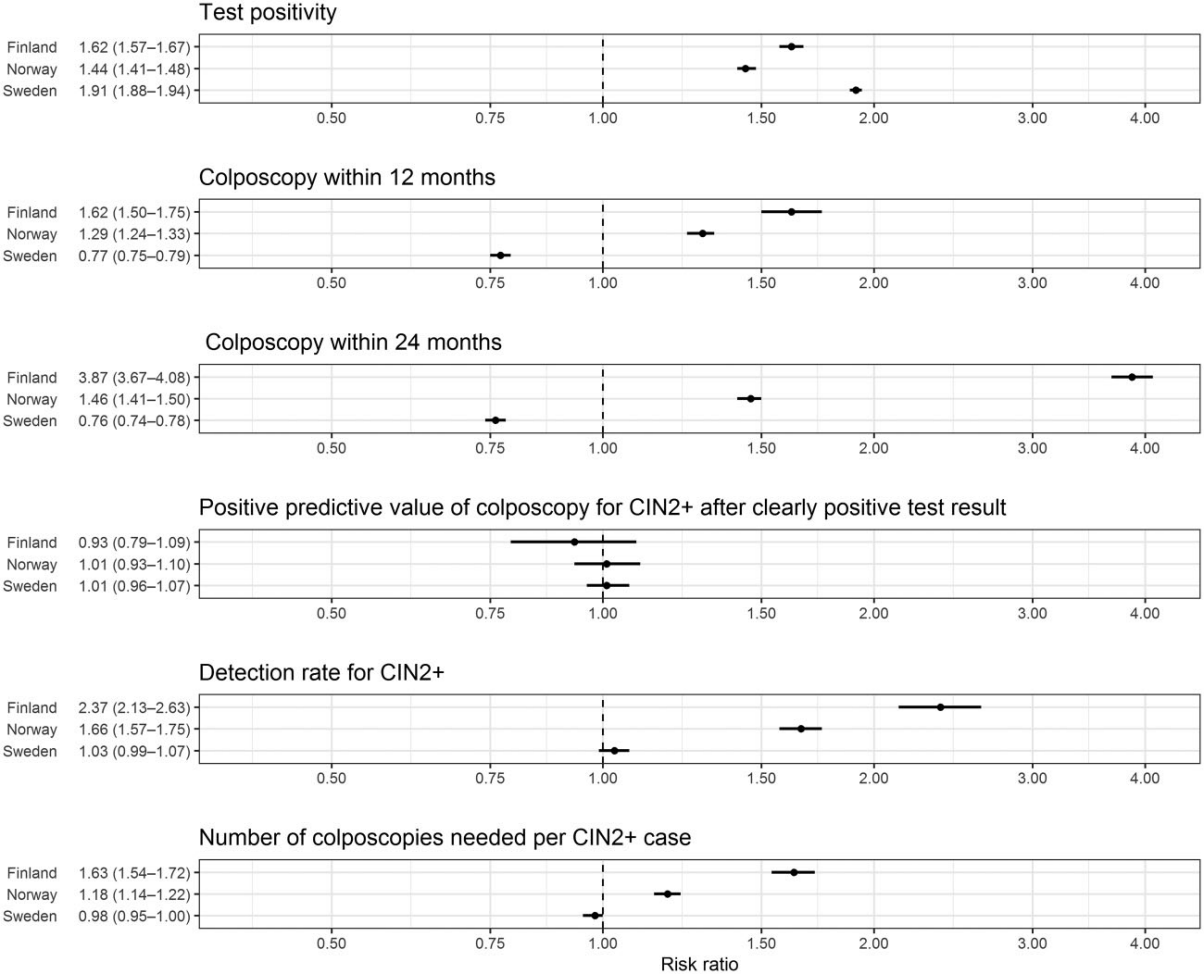
Age-adjusted RRs for different outcomes in HPV testing compared with cytology in 30- to 64-year-old women in 2015–17.

Figure 1 shows age-adjusted RR by country for different outcomes of screening. Test positivity was higher for HPV than cytology for all countries (RR varied between 1.44 and 1.91). Colposcopies within 12 or 24 months were more likely with HPV testing in Finland and Norway, but the RR was around 0.8 (95% CI varied between 0.74 and 0.79) for Sweden.

PPV of colposcopy after a clearly positive test result was similar for both test methods in all included countries. The relative DR for CIN2+ was highest in Finland (2.37, 95% CI = 2.13–2.63) and slightly lower in Norway (RR 1.66, 95% CI = 1.57–1.75) while the difference between test methods in Sweden was not statistically significant (RR 1.03, 95% CI = 0.99–1.07). The relative number of colposcopies needed per CIN2+ case in HPV-based testing compared with cytology was higher in Finland (RR 1.63, 95% CI = 1.54–1.72) and Norway (RR 1.18, 95% CI = 1.14–1.22) but almost equal in Sweden (RR 0.98, 95% CI = 0.95–1.00). The sensitivity analysis for women aged 34–64 years did not alter RRs.

## Discussion

### Key findings 

The effects of implementing HPV screening varied by country as different screening algorithms were implemented. The results indicate different balances of benefits and harm, particularly for cytological screening. However, these balances seem to converge in the early HPV screening implementation phase studied. The number of colposcopies was higher in HPV-based screening compared with cytology-based screening in Finland and Norway but lower in Sweden. The DR was higher in HPV-based screening in Finland and Norway at the cost of increased numbers of colposcopies. In Sweden, both the DR for CIN2+ and the number of colposcopies needed for finding a CIN2+ case did not differ between test methods.

### Interpretation of findings

Colposcopy rates in HPV testing compared with cytology were higher in Finland and Norway, which is in line with prior studies.^19–21^ In Sweden, however, the colposcopy rates were lower in HPV testing and is explained by a different screening algorithm where HPV positive and cytology-negative women were tested again after a 3-year interval while repeat testing occurred earlier in Finland and Norway. There were also a higher proportion of colposcopies in Sweden and Norway after a normal test result in cytology-based testing compared with HPV testing. This might indicate clinical testing and colposcopy referrals if a test was taken based on symptoms, which our method has classified as cytology-based primary test. An Australian modelling study^22^ estimated that the number of colposcopies would increase by 34% during the first screening round when switching to HPV-based screening, which is a modest increase compared with the relative colposcopy rate of 3.9 in Finland at 24 months in our study. The results indicate that performance of cytology varies highly between the cytology screening programmes, which will impact any comparison of HPV and cytology-based screening performance.

The PPVs after clearly positive results were similar for both methods, which is to be expected as ASC-H+/AGC+ cytology defined test positivity regardless of test method. Our analysis also showed that in Finland AGC-NOS result of cytology was only rarely followed up with colposcopy in the primary testing episode during the study period.

The DRs were higher for HPV-based screening compared with cytology in Finland and Norway while the DR was similar in Sweden between test methods. Higher DRs have also been reported in many European countries implementing HPV-based screening.^23^ Our results are in line with the Finnish randomized HPV trial, which reported a hazard ratio of 1.71 for CIN2 lesions compared with cytology.^24^ The results of similar DRs between test methods in Sweden are also in line with a Swedish trial that reported no statistically significant differences in DR of CIN2+ between HPV tests and cytology.^25^ The DR in this study was lower in Finland compared with other Nordic countries, which may partly be due to a lower background risk of precancerous lesions as the incidence of cervical cancer and referral rate to colposcopy is lower compared with other Nordic countries. There might also be differences in the diagnostic criteria. Guidelines on diagnostic referrals are also not always followed as exemplified by the number of colposcopies after normal test results. Furthermore, the study population in Finland was limited to invitational screening population whereas in other countries the data also included population subject to opportunistic and diagnostic testing. Missing data from other sources in Finland may have lowered the estimated colposcopy and DRs in Finland as ∼60% of tests are taken outside the screening programme.^26^ However, comparison between cytology and HPV testing is valid since there is no reason to assume that the extent of opportunistic testing differed between the test methods.

The number of colposcopies per CIN2+ case can be used as a proxy for the balance of benefits and harms of screening. Even though there were differences between Nordic countries in the number of colposcopies needed for finding a CIN2+ case, in most studied settings that ratio (ranging from 2.3 to 3.1 with the exception of 5.0 for HPV testing in Finland) was in line with numbers reported in the Dutch programme (1.9 and 2.7 for cytology and HPV testing, respectively).^27^ The number was significantly lower than in the ATHENA trial from USA where the number of colposcopies needed for a CIN2+ case was 7–8 depending on screening algorithm and only reported for two groups of women over 25 years and over 30 years.^28^ This difference is likely explained by the screening algorithm in the ATHENA trial as the colposcopy referral threshold was lower than in the Nordic screening programmes.

Overdiagnosis of precancerous lesions in screening should also be considered. Approximately half of CIN2 lesions are estimated to regress and only one in five to progress to CIN3 or worse within 2 years.^29^ As HPV testing increases the DR of these lesions, it is important to reconsider the alternative clinical management options, such as active surveillance. Overtreatment should be avoided since treatment of precancerous lesions have potential short- and long-term complications, such as preterm birth.^30^ In this study, the endpoints for PPV and DR were defined at CIN2+ because the distinction between CIN2 and CIN3 was not available from all countries for all years. Incidentally, our study follows the updated World Health Organization classification where CIN2 and CIN3 are grouped into HSIL.^31^

Our study shows that differences in the algorithms of screening also cause divergent changes when implementing HPV-based screening. In Finland and Norway, the number of colposcopies and DR increased while in Sweden, the number of colposcopies even decreased while DR remained at same level compared with cytology. Using partial or extended HPV genotyping when triaging HPV-positive women and having a higher referral threshold for less oncogenic HPV types is a potential strategy for reducing colposcopy referrals while maintaining high DRs.^32^ Triage algorithms utilizing genotyping and viral load could also reduce overdiagnosis as genotypes differ in their likelihood to progress to invasive cancer.^33^ Partial genotyping was implemented in Norway from 1 July 2018 onwards, where only women tested positive for genotypes 16 or 18 with low-grade abnormal cytology were referred to diagnostic confirmation. The Swedish screening programme has taken a step further and will implement extended genotyping in the routine programme by classifying HPV genotypes to high risk (HPV types 16, 18 and 45), medium risk (HPV types 31, 33, 52 and 58) and low risk (other hrHPV types) and apply different management pathways based on this grouping. Overdiagnosis within women under 29 years will be limited by referring to colposcopy only women with hrHPV positivity and abnormal cytology in the triage test.

Our results are mostly from the initial round of HPV screening and the colposcopy rates are likely to decline in the subsequent screening rounds as the more sensitive test finds prevalent HPV infections and precancerous lesions that are treated. As health care resources are limited, it is also crucial that screening continues to align with value-based health system approach.^34^ Surveillance of HPV testing in subsequent screening rounds is needed to ensure cost-effectiveness and optimal balance between benefits and harms.

### Strengths and limitations

The main strength of our study is the use of data registered in comprehensive national databases. The dataset was large, consisting of nearly 3 million test episodes of which over 400 000 were HPV test episodes. We also linked test episodes and histological data from the same individual together and avoided counting tests from same women multiple times. We harmonized the data between registers using identical procedures to ensure comparability.

The biggest limitation of our study is that the data are from routine screening setting where the implementation of HPV-based screening was not randomized in all countries, and we could not control for other potential differences. For Finland, the lack of data outside organized programme may lead to artificially low colposcopy and DRs as all histological diagnoses were not included. The length of the study period did not allow analysis of hrHPV persistence and subsequent colposcopy referrals at repeat testing in Sweden.

## Conclusions

The effects of implementing HPV screening varied between countries and were dependent on screening algorithms used. HPV-based screening increases colposcopy rates mainly through referrals from increased repeat testing and DR is therefore significantly higher compared with cytology. Monitoring of these indicators in the subsequent rounds of HPV-based screening remains essential.

## Supplementary Material

ckad225_Supplementary_Data

## Data Availability

Indicators based on the aggregated data are available at the Nordscreen project website nordscreen.org. Other access to data is available upon reasonable request by contacting the corresponding author. Key points The effects of implementing HPV-based screening varied considerably by country. Colposcopy referrals were increased mainly through repeat testing. Detection rates of precancerous lesions also increased in HPV-based screening. Monitoring of subsequent rounds in HPV-based screening remains essential.
