# Supplementary material for: Divergent effects of switching from cytology to HPV-based screening in the Nordic countries
Source: Eur J Public Health. 2024 Jan 22;34(2):354–60. doi: 10.1093/eurpub/ckad225 (PMC10990554; doi:10.1093/eurpub/ckad225)
Supplement: ckad225_Supplementary_Data [file ckad225_supplementary_data.pdf]

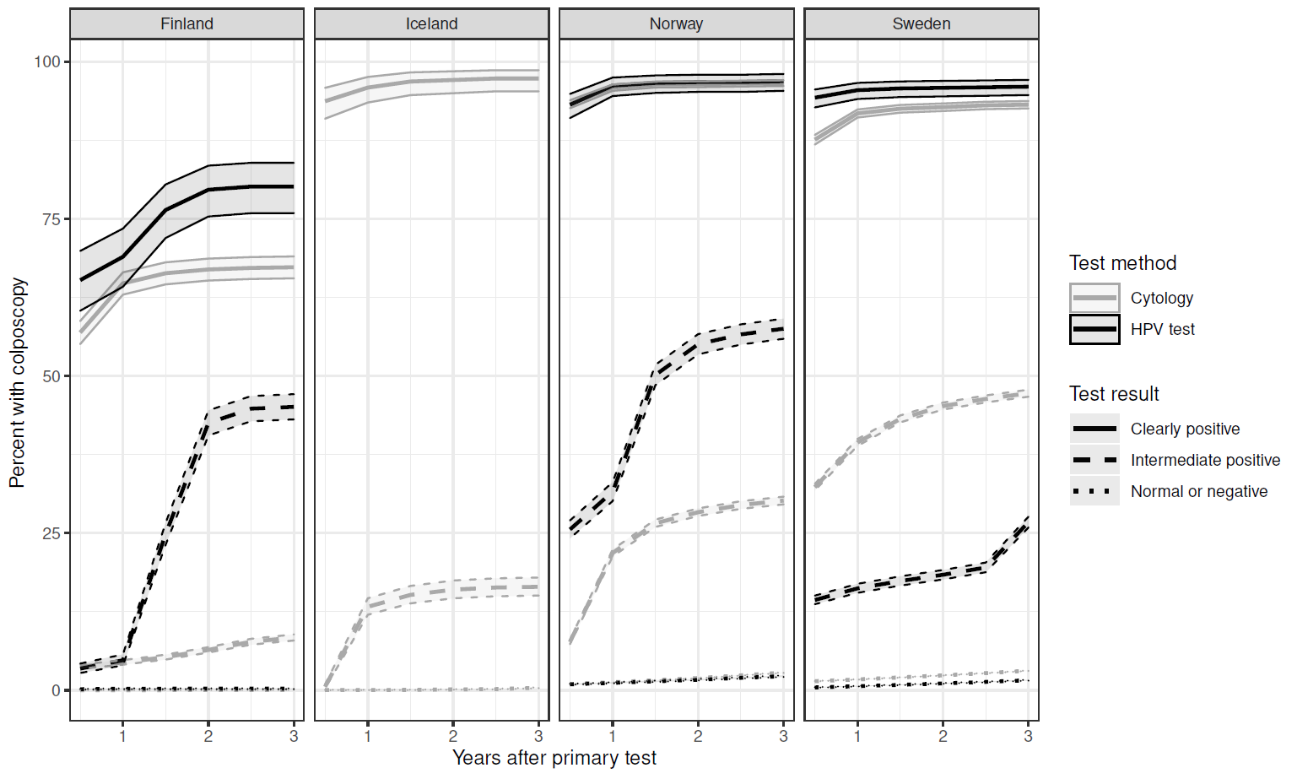


**Supplementary Figure 1.** Proportion of women with colposcopy within three years after primary test during 2015-2017. Follow-up time varies from 6 months to 36 months. The shaded areas represent 95% confidence intervals**.**

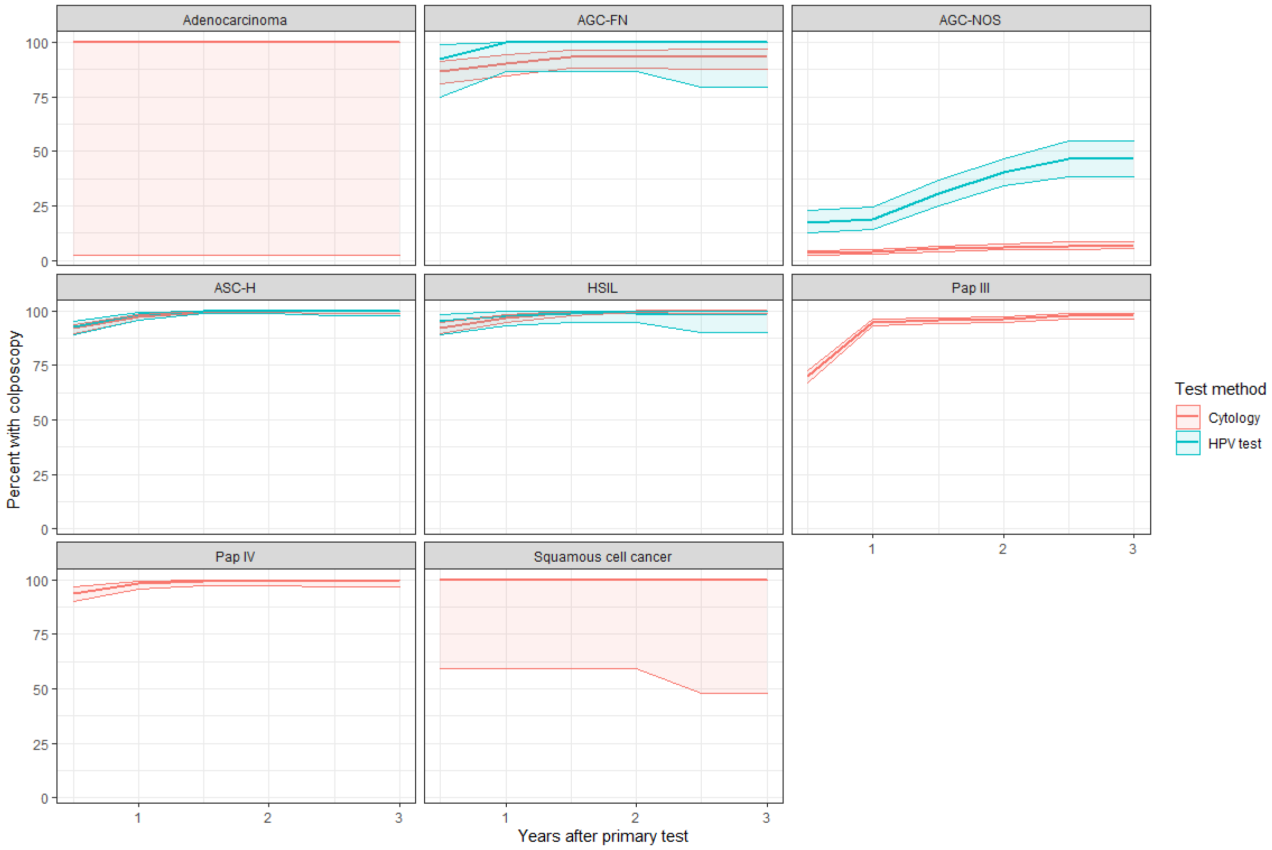


**Supplementary Figure 2.** Proportion of women with colposcopy within three years after a clearly positive primary test in Finland during 2015-2017. Follow-up time varies from 6 months to 36 months. The shaded areas represent 95% confidence intervals.
